# Fevers and Some Other Diseases in South Florida

**Published:** 1890-12

**Authors:** N. A. Williams

**Affiliations:** Macon, Fla.


### FEVERS AND SOME OTHER DISEASES IN SOUTH FLORIDA.

BY N. A. WILLIAMS, M. D., MACON, FLA.

In this climate most fevers are ushered in slowly and insidiously, and often the patient will not cease to attend his business pursuits for a number of days before taking his bed. The patient, in many instances, though with light fever, say one or two degrees above normal and almost continuously, may not be put to the necessity of confinement to his room or bed, but with light treatment prescribed by himself continues at his post of duty, not losing a day till cured. This must be peculiar to our climate which is remarkable for its healthfulness, which statistical reports corroborate. The Peninsular portion of South Florida is a wonderful arm of land thrust down into the sea more than 300 miles in length and in many places but little more than 100 miles in width. These salt breezes from the East and from the West are often curative of incipient lung troubles without a dose of medicine. The danger to the patient is that the fever being of a light and apparently insignificant type leads him astray for many days without any treatment whatever, and thus diseases of a graver character are strongly invited, and he falls a ready prey to dropsy, albuminuria and in some instances urenic taxemia. However, with these complications he is much more amenable to curative treatment than in any locality of which I have a knowledge, with the proviso of confining this knowledge to the Southern States. The prevailing fevers here are of a mild, intermittent, or remittent type. Typhoid fever is rare in South Florida, and so far as my knowledge and experience extends, which covers a fair practice of eight years, is quite amenable to successful treatment.

